# New records of bee flies (Diptera, Bombyliidae) from Cuatro Ciénegas, Coahuila, Mexico

**DOI:** 10.3897/zookeys.422.7598

**Published:** 2014-07-03

**Authors:** Omar Ávalos-Hernández, Joel Kits, Marysol Trujano-Ortega, Uri Omar García-Vázquez, Zenón Cano-Santana

**Affiliations:** 1 Museo de Zoología, Facultad de Ciencias, UNAM, Apdo. Postal 70-399. México 04510 D.F. México; 2 6864 Gallagher Rd., Ottawa, Canada; 3 Departamento de Ecología y Recursos Naturales, Facultad de Ciencias, UNAM Circuito Exterior s/n, Ciudad Universitaria, México 04510 D.F. México

**Keywords:** Biodiversity, distribution expansion, Nearctic region, desert fauna

## Abstract

Forty one new records of species of Bombyliidae are reported for Coahuila in northeastern Mexico. Nine of these species are reported for the first time for the country. The specimens were collected in the Cuatro Ciénegas Basin and Sierra La Madera mountains during 2007–2013. The modified distributions of species are discussed. The gaps in the distribution of many species suggest an undersampling of this group of insects in the north of Mexico.

## Introduction

The bee flies (Bombyliidae) belong to the superfamily Asiloidea and are the eighth most diverse family within Diptera with 5382 described species (Pape et al. 2011). All species of Bombyliidae are parasitoids, hyperparasitoids or predators of immature stages of Coleoptera, Hymenoptera, Lepidoptera, Orthoptera, Neuroptera, and Diptera (Yeates and Greathead 1997, Boesi et al. 2009). Unlike most other taxa, bee flies are most abundant and diverse in arid and semiarid portions of the world (Hull 1973, Evenhuis 1989). In the immature stages these insects function as a natural control for populations of other insects and as adults are efficient pollinators (Motten et al. 1981, Kearns 2001).

Some faunistic studies have been completed including Bombyliidae in Mexico (Rodríguez-Ortuño 1989, Ávalos-Hernández 2007), but the northern region of the country is poorly known for this family. Although Evenhuis and Greathead (1999) list 15 species of Bombyliidae for Coahuila, species richness in this state is probably higher as suggested by the richness of surrounding Mexican states with similar or even smaller size and similar ecosystems (e.g., Nuevo León, 37 species; Durango, 41 species) and of Texas (171 species), the nearest USA state.

Cuatro Ciénegas Basin in the northeast of Coahuila is especially interesting because of its geological history and the presence of water ponds and gypsum dunes, which create a different environment from the surrounding areas. The basin was a shallow sea from the Pangea breakup until the Eocene, 40 Ma, when the Sierra Madre Oriental in the east of Mexico rose isolating the Basin from the Atlantic Ocean (Souza et al. 2012). The physiology of Cuatro Ciénegas bacteria is similar to that of marine species, with which they are closely related (Souza et al. 2006). According to Moreno-Letelier et al. (2012) this evidence indicates that some water was kept trapped in the Basin when the ocean retreated giving the basin unique characteristics. These characteristics produced a high number endemism for vertebrates and prokaryotes in Cuatro Ciénegas (Souza et al. 2006, 2012).

The present study is the first known long-term systematic sampling of Diptera in Cuatro Ciénegas. The objective of this project is to complete the list of species of Bombyliidae in the basin and surrounding mountains. In this paper, 41 new species-level records for Coahuila from Cuatro Ciénegas are presented, including nine new records for Mexico. The modified distributions of the species are discussed.

## Methods

**Figure 1.**
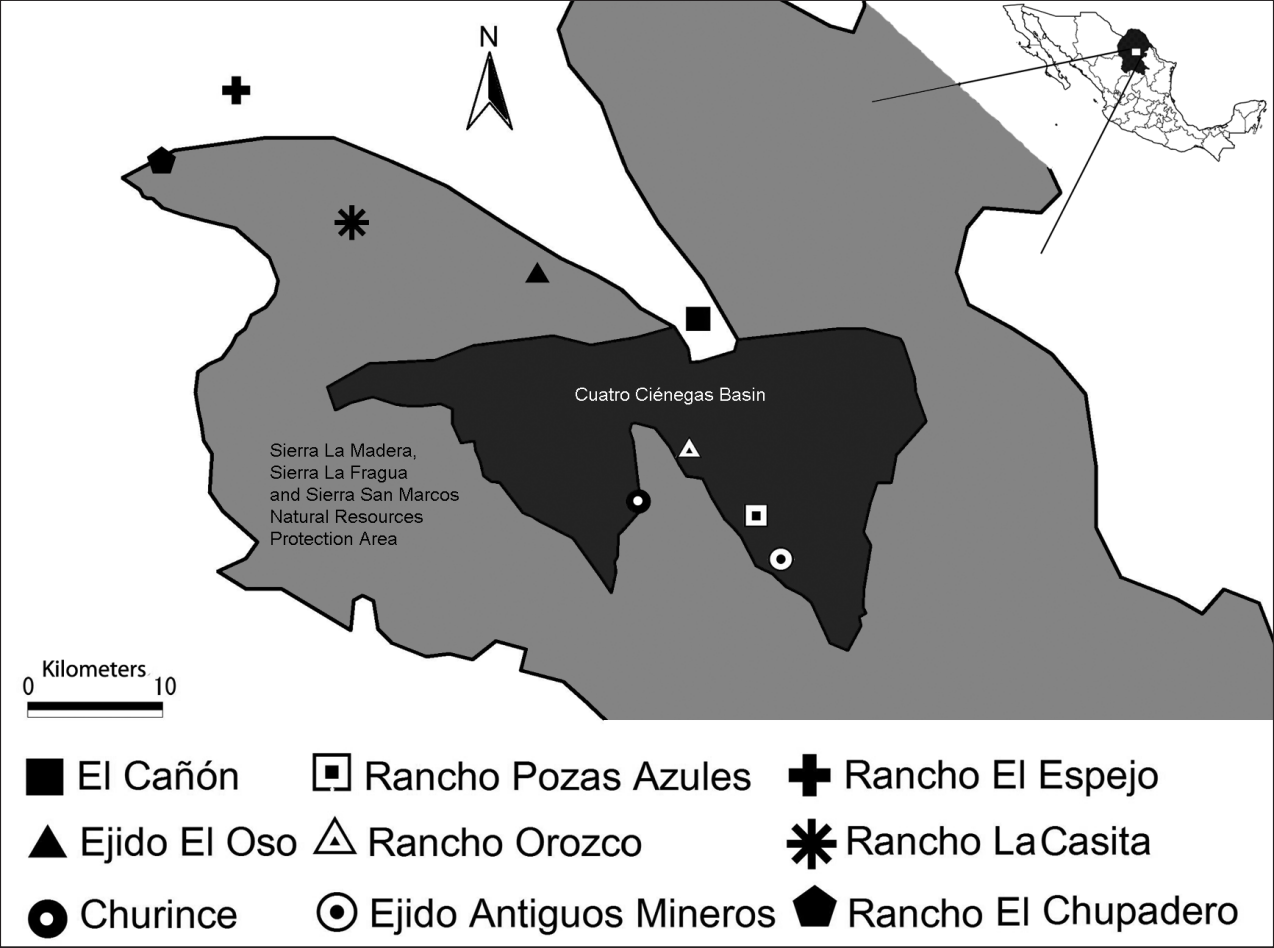
Field work sites. Cuatro Ciénegas basin is located in Coahuila at northeast of Mexico. Sierra La Madera is located at northwest of the basin.

Beeflies were collected at nine sites from Cuatro Ciénegas Basin and Sierra La Madera within the Municipality of Cuatrociénegas (Figure 1). Abbreviations for study sites (Table 1) are used throughout. Samplings were performed during 2007-2013, using aerial net and a Malaise trap. The Malaise trap had white polyester netting, was square in configuration, 210 cm tall and 120 cm wide and the collecting head located at the top. Trap was set from 9:00 to 17:00 when weather conditions allowed it. To avoid damage to the specimens no killing agent was used, insects were extracted at the end of the day. Specimens were pinned and labeled. Generic identification was carried out under a stereomicroscope according to the keys by Hall (1981b) and Kits et al. (2008). Species were identified by the first and second authors with specialized keys for each genus and comparison with museum specimens, keys used for identification of each genera are specified below. Taxonomic classification and distribution data are based on Evenhuis and Greathead (1999) and host data are based on Hull (1973), if not indicated otherwise. Distribution gaps are suggested as disjunct distribution patterns or the result of under sampling by comparing the location of records in Mexico with those in the southern states of the USA. All specimens are deposited in the Colección Nacional de Insectos (Instituto de Biología, Universidad Nacional Autónoma de México; CNIN-IBUNAM).

**Table 1.** Field work sites in Cuatro Ciénegas. Vegetation according to Pinkava (1979).

	Site (Code)	Location	Altitude (m)	Vegetation
1	Churince (CHU)	26°50'30"N, 102°08'10"W	770	Gypsum dunes; sedges and marshes; mezquital, halophile
2	Rancho Orozco (ROR)	26°52'18"N, 102°05'17"W	740	Sedges and marshes; mezquital; halophile
3	Rancho Pozas Azules (RPA)	26°49'39"N, 102°01'24"W	710	Sedges and marshes; mezquital; halophile
4	Ejido Antiguos Mineros (EAM)	26°46'58"N, 102°00'20"W	725	Sedges and marshes; mezquital; halophile
5	El Cañón (ECA)	27°00'34"N, 102°04'42"W	780	Mezquital; desert scrub
6	Ejido El Oso (EEO)	27°03'08"N, 102°13'35"W	1085	Desert scrub; chaparral
7	Rancho El Espejo (REE)	27°13'19"N, 102°30'19"W	1425	Desert scrub; chaparral
8	Rancho El Chupadero (REC)	27°10'07"N, 102°34'26"W	1790	Desert scrub; chaparral; Pine-Oak forest
9	Rancho La Casita (RLC)	27°06'45"N, 102°23'40"W	1630	Desert scrub; chaparral; Pine-Oak forest

## Results

A total of 41 new species-level records are presented for the state of Coahuila. Nine of these 41 species are recorded for the first time in Mexico, being their most southern records. Of the 15 species previously listed for Coahuila, two were collected during this study: *Heterostylum robustum* (Osten Sacken, 1877) (Material collected: CHU: Apr (1 M), Sep (1 M); EAM: Mar (2 M), Sep (1 F), Jun (2 F), Jul (1 F), Oct (1 F); ROR: Apr (1 F, 3 M), May (1 F, 2 M), Jul (3 F), Sep (1 F); RPA: Apr (1 F, 1 M), Jul (2 F, 2 M), Sep (1 M), Oct (2 M)); and *Anastoechus melanohalteralis* Tucker, 1907 (Material collected: EAM: Oct (1 M); ECA: Oct (1 F, 1M); ROR: Oct (7 F, 6 M); RPA: Sep (1 M)).

New records of the species included in this paper are from 17 genera for which modern revisions are available. Six taxa of *Hemipenthes* (3), *Lordotus* (1), *Paravilla* (1) and *Rhynchanthrax* (1) could not be identified accurately, being probably undescribed species. Identification of species in another 10 genera found in the study (e.g. *Villa*, *Chrysanthrax*, and *Exoprosopa*) is difficult and unreliable. The number of morphospecies and specimens collected of these genera are presented in Table 2. Six species of *Tmemophlebia* (1), *Geron* (1), *Exoprosopa* (3) and *Villa* (1) previously listed for Coahuila were probably collected but specimens of these genera are still being identified. Taxonomic work will continue, updates of the species list and descriptions of the new taxa will be published in subsequent papers.

**Table 2.** Updated list of genera and species of Bombyliidae in Coahuila (* species not collected in this study, but recorded previously in Coahuila; ** species most likely collected in this study, but identification not yet certain).

Subfamily, genus and species name	New record	Unidentifiable material
**PHTHRIINAE**		
***Neacreotrichus* Cockerell**		
* *Neacreotrichus consors* (Osten Sacken, 1887)		
***Poecilognathus* Jaennicke**	Coahuila	1 morphospecies, 3 specimens
***Relictiphthiria* Evenhuis**		
* *Relictiphthiria psi* (Cresson, 1919)		
***Tmemophlebia* Evenhuis**		1 morphospecies, 21 specimens
** *Tmemophlebia coquilletti* (Johnson, 1902)		
**TOXOPHORINAE**		
***Geron* Meigen**		2 morphospecies, 194 specimens
** *Geron holosericeus* Walker, 1849		
***Systropus* Wiedemann**	Coahuila	1 morphospecies, 5 specimens
***Toxophora* Meigen**	Coahuila	
*Toxophora maxima* Coquillett, 1886	Coahuila	
*Toxophora virgata* Osten Sacken, 1877	Coahuila	
**BOMBYLIINAE**		
***Anastoechus* Osten Sacken**		
*Anastoechus melanohalteralis* Tucker, 1907		
***Bombylius* Linnaeus**		
*Bombylius (Bombylius) frommerorum* Hall & Evenhuis, 1980	Coahuila	
* *Bombylius (Bombylius) sylphae* Evenhuis, 1984		
* *Bombylius (Parabombylius) aleophilus* (Hall & Evenhuis, 1981)		
* *Bombylius (Parabombylius) coahuilensis* (Hall & Evenhuis, 1981)		
* *Bombylius (Parabombylius) paradoxus* (Hall & Evenhuis, 1981)		
* *Bombylius (Parabombylius) syndesmus* (Coquillett, 1894)		
***Conophorus* Meigen**	Coahuila	1 morphospecies, 3 specimens
***Heterostylum* Macquart**		
*Heterostylum croceum* Painter, 1930	Mexico	
*Heterostylum robustum* (Osten Sacken, 1877)		
***Lordotus* Loew**	Coahuila	1 morphospecies, 38 specimens
*Lordotus diplasus* Hall, 1954	Coahuila	
*Lordotus divisus* Cresson, 1919	Coahuila	
*Lordotus perplexus* Johnson & Johnson, 1959	Coahuila	
***Triploechus* Edwards**	Coahuila	
*Triploechus novus* (Williston, 1893)	Coahuila	
**LOMATIINAE**		
***Ogcodocera* Macquart**	Coahuila	
*Ogcodocera analis* Williston, 1901	Coahuila	
**TOMOMYZINAE**		
***Paracosmus* Osten Sacken**	Coahuila	
*Paracosmus (Paracosmus) morrisoni* Osten Sacken, 1887	Coahuila	
**ANTHRACINAE**		
***Anthrax* Scopoli**	Coahuila	
*Anthrax atriplex* Marston, 1970	Coahuila	
*Anthrax cybele* (Coquillett, 1894)	Mexico	
*Anthrax georgicus* Macquart, 1834	Coahuila	
*Anthrax irroratus* Say, 1823	Coahuila	
*Anthrax oedipus* Fabricius, 1805	Coahuila	
*Anthrax pauper* (Loew, 1869)	Mexico	
*Anthrax seriepunctatus* (Osten Sacken, 1886b)	Coahuila	
***Aphoebantus* Loew**	Coahuila	4 morphospecies, 236 specimens
***Chrysanthrax* Osten Sacken**	Coahuila	6 morphospecies, 240 specimens
***Dipalta* Osten Sacken**	Coahuila	
*Dipalta serpentina* (Osten Sacken, 1877)	Coahuila	
***Exoprosopa* Macquart**		9 morphospecies, 395 specimens
** *Exoprosopa aztec* Painter & Painter, 1969		
** *Exoprosopa butleri* Johnson & Johnson, 1958		
** *Exoprosopa dorcadion* Osten Sacken, 1877		
***Hemipenthes* Loew**	Coahuila	3 morphospecies, 146 specimens
*Hemipenthes jaennickeana* (Osten Sacken, 1886a)	Coahuila	
*Hemipenthes lepidota* (Osten Sacken, 1886b)	Coahuila	
*Hemipenthes scylla* (Osten Sacken, 1887)	Coahuila	
*Hemipenthes sinuosa* (Wiedemann, 1821)	Coahuila	
***Lepidanthrax* Osten Sacken**	Coahuila	
*Lepidanthrax arizonensis* Hall, 1976	Mexico	
*Lepidanthrax disiunctus* (Wiedemann, 1830)	Coahuila	
*Lepidanthrax hesperus* Hall, 1976	Coahuila	
*Lepidanthrax hyposcelus* Hall, 1976	Coahuila	
*Lepidanthrax proboscideus* (Loew, 1869)	Coahuila	
***Ligyra* Newman**	Coahuila	1 morphospecies, 2 specimens
***Neodiplocampta* Curran**	Coahuila	
*Neodiplocampta (Neodiplocampta) miranda* Hull & Martin, 1974	Coahuila	
***Paravilla* Painter**	Coahuila	1 morphospecies, 48 specimens
*Paravilla edititoides* (Painter, 1933)	Coahuila	
*Paravilla flavipilosa* (Cole, 1923)	Coahuila	
*Paravilla parvula* Hall, 1981a	Coahuila	
*Paravilla separata* (Walker, 1852)	Mexico	
***Poecilanthrax* Osten Sacken**	Coahuila	
*Poecilanthrax effrenus* (Coquillett, 1887)	Coahuila	
*Poecilanthrax fasciatus* Johnson & Johnson, 1957	Mexico	
*Poecilanthrax hyalinipennis* Painter & Hall, 1960	Mexico	
*Poecilanthrax poecilogaster* (Osten Sacken, 1886b)	Coahuila	
***Rhynchanthrax* Painter**	Coahuila	1 morphospecies, 70 specimens
*Rhynchanthrax capreus* (Coquillett, 1887)	Mexico	
*Rhynachantrax texanus* (Painter, 1933)	Coahuila	
***Thyridanthrax* Osten Sacken**	Coahuila	
*Thyridanthrax pallidus* (Coquillett, 1887)	Mexico	
*Thyridanthrax selene* (Osten Sacken, 1886b)	Coahuila	
***Villa* Lioy**		9 morphospecies, 115 specimens
** *Villa fumicosta* Painter & Painter, 1962		
***Xenox* Evenhuis**	Coahuila	
*Xenox xylocopae* (Marston, 1970)	Coahuila	

A total of 28 genera were found during this study, of which 21 are new records for the state. Two genera previously listed for Coahuila (*Neacreotrichus* and *Relictiphthiria*) were not found in Cuatro Ciénegas area. With the new records presented here, the list of bee fly species in Coahuila increases to 56 (Table 2).

### Subfamily Toxophorinae

#### Genus Toxophora

Taxon classificationAnimaliaDipteraBombyliidae

Meigen

##### Remarks.

*Toxophora* is distributed worldwide, being more diverse in the Afrotropical and Palearctic regions. Mexico's fauna includes three Neotropical species and five Nearctic species. All Nearctic species of Mexico were distributed in the western half of the country. These two new records represent the first of this genus in Coahuila and the most eastern distribution of the Nearctic species in the country. The New World species of this genus were keyed using Cunha et al. (2011).

#### Toxophora maxima

Taxon classificationAnimaliaDipteraBombyliidae

Coquillett, 1886

Figure 2a, b

##### Material examined.

CHU: Jul (1 M); EEO: Jul (2 F, 2 M), Oct (1 F, 3 M).

##### Known Nearctic records.

Mexico (Baja California, Baja California Sur, Coahuila); USA (Arizona, California, Idaho, Kansas, New Mexico, Oklahoma, Oregon, Texas).

##### Comments.

In Mexico *Toxophora maxima* was only known from Baja California Peninsula and now Coahuila. This apparent gap in its distribution is probably due to undersampling. Sampling of the intermediate zones is necessary to know if these populations form a continuous unit as they do in the southern states of USA.

**Figure 2.**
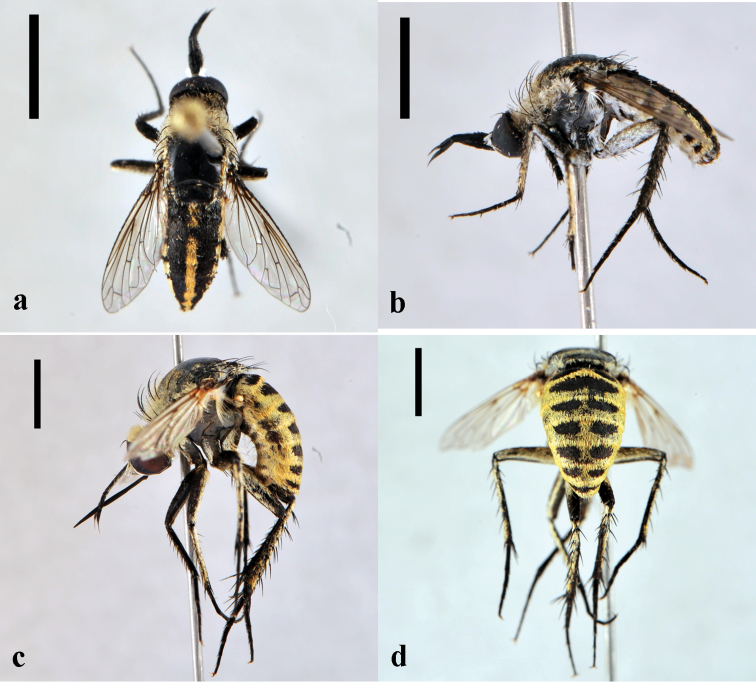
*Toxophora*. *Toxophora maxima*, male (CNIN 1115) **a** dorsal view **b** lateral view; *Toxophora virgata*, male (CNIN 1109) **c** lateral view **d** posterior view. All scale bars: 3 mm.

#### Toxophora virgata

Taxon classificationAnimaliaDipteraBombyliidae

Osten Sacken, 1877

Figure 2c, d

##### Material examined.

EAM: Jun (1 F, 1 M), Jul (1 F); CHU: Aug (1 M), Oct (1 M); EEO: Jul (1 M), Oct (1 F, 1 M); RLC: Jun (1 M); ROR: Apr (1 F, 2 M); RPA: Oct (1 F).

##### Known Nearctic records.

Mexico (Baja California Sur, Coahuila, Sonora); USA (Arizona, California, Colorado, Georgia, Idaho, Nevada, New Mexico, Oklahoma, Texas, Utah).

##### Known hosts.

*Odynerus* sp. (Vespidae); *Stenodynerus toltecus* Saussure (Vespidae).

##### Comments.

This species is present in the all southwestern states of the USA and northwest of Mexico. This is the first record in the northeast of Mexico. The species is probably also present in Chihuahua, between Sonora and Coahuila.

### Subfamily Bombyliinae

#### Genus Bombylius

Taxon classificationAnimaliaDipteraBombyliidae

Linnaeus

##### Remarks.

With 278 described species, *Bombylius* is the second most diverse genus of Bombyliidae. It has a worldwide distribution being especially diverse in the Palearctic and Nearctic regions. One endemic species is present in Coahuila: *Bombylius (Parabombylius) coahuilensis* (Hall & Evenhuis, 1981). Four other species are reported for the state: *Bombylius sylphae* Evenhuis, 1984, *Bombylius aleophilus* (Hall & Evenhuis, 1981), *Bombylius paradoxus* (Hall & Evenhuis, 1981), *Bombylius syndesmus* (Coquillett, 1894). A review with identification keys for Nearctic species is presented in Hall and Evenhuis (1980), later Evenhuis (1984) revised and present keys for the *comanche* group of America.

#### Bombylius (Bombylius) frommerorum

Taxon classificationAnimaliaDipteraBombyliidae

Hall & Evenhuis, 1980

Figure 3

##### Material examined.

EEO: Aug (1 M), Oct (1 F).

##### Known Nearctic records.

Mexico (Chihuahua, Coahuila); USA (Arizona, New Mexico, Texas).

##### Comments.

This species is restricted to the southwest of the USA and north of Mexico.

**Figure 3.**
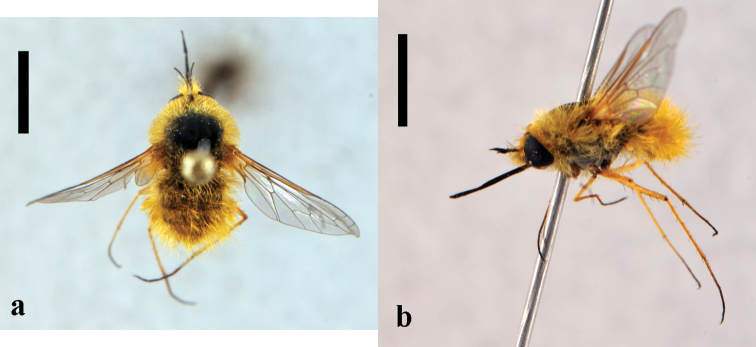
*Bombylius (Bombylius) frommerorum*, female (CNIN 772) **a** dorsal view **b** lateral view. All scale bars: 3 mm.

#### Genus Heterostylum

Taxon classificationAnimaliaDipteraBombyliidae

Macquart

##### Remarks.

The genus is only present in Nearctic and Neotropical regions. Although not as diverse as other genera (only 12 species), specimens from some species are abundant in the field. *Heterostylum robustum* was previously known from Coahuila and was collected during this study. This species is distributed from Canada to central Mexico. There are two revisions for this genus that contains identification keys, one by Hall and Evenhuis (1980) and the more recent by Cunha et al. (2007).

#### Heterostylum croceum

Taxon classificationAnimaliaDipteraBombyliidae

Painter, 1930

Figure 4

##### Material examined.

REE: Apr (1 F).

##### Known Nearctic records.

Mexico (Coahuila); USA (Colorado, Kansas, Missouri, New Mexico, Texas).

##### Comments.

*Heterostylum croceum* is recorded for the first time in Mexico; previously known from the southern-central United States. Hall and Evenhuis (1980) suggest that *Heterostylum croceum* may be related to *Heterostylum engelhardti* Painter, 1930 or even be a subspecies of that taxon, *Heterostylum croceum* is the eastern form and *Heterostylum engelhardti* the western form (Arizona, California, Texas, Utah) although both species are present in Texas. Cunha et al. (2007) comment that *Heterostylum engelhardti* can be distinguished by the presence of white to very pale yellow hair and brown-tipped hairs on the abdomen compared with the darker yellow hairs in *Heterostylum croceum*.

**Figure 4.**
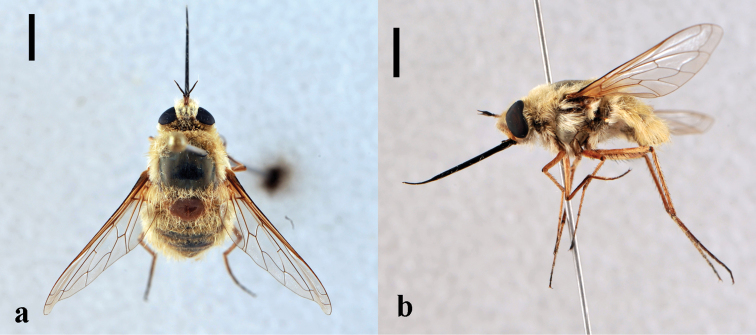
*Heterostylum croceum*, female (CNIN 858) **a** dorsal view **b** lateral view. All scale bars: 3 mm.

#### Genus Lordotus

Taxon classificationAnimaliaDipteraBombyliidae

Loew

##### Remarks.

Most of the 29 species in this exclusively Nearctic genus are distributed in the southwest of the USA and north of Mexico, although eight species are present in the northwest of the USA (*Lordotus apicula* Coquillet, 1887; *Lordotus bipartitus* Painter, 1940; *Lordotus diversus* Coquillett, 1891; *Lordotus gibbus* Loew, 1863; *Lordotus miscellus* Coquillett, 1887; *Lordotus pulchrissimus* Williston, 1893; *Lordotus striatus* Painter, 1940; *Lordotus zona* Coquillett, 1887). The three species present in Coahuila are also found in California; their distribution probably includes all northern states of Mexico. Hall (1954) and Hall and Evenhuis (1982) present reviews of the genus and keys to the species.

#### Lordotus diplasus

Taxon classificationAnimaliaDipteraBombyliidae

Hall, 1954

Figure 5a, b

##### Material examined.

CHU: Sep (2 M); RLC: Sep (2 M); RPA: Sep (1 F).

##### Known Nearctic records.

Mexico (Coahuila, Zacatecas); USA (Arizona, California, New Mexico).

**Figure 5.**
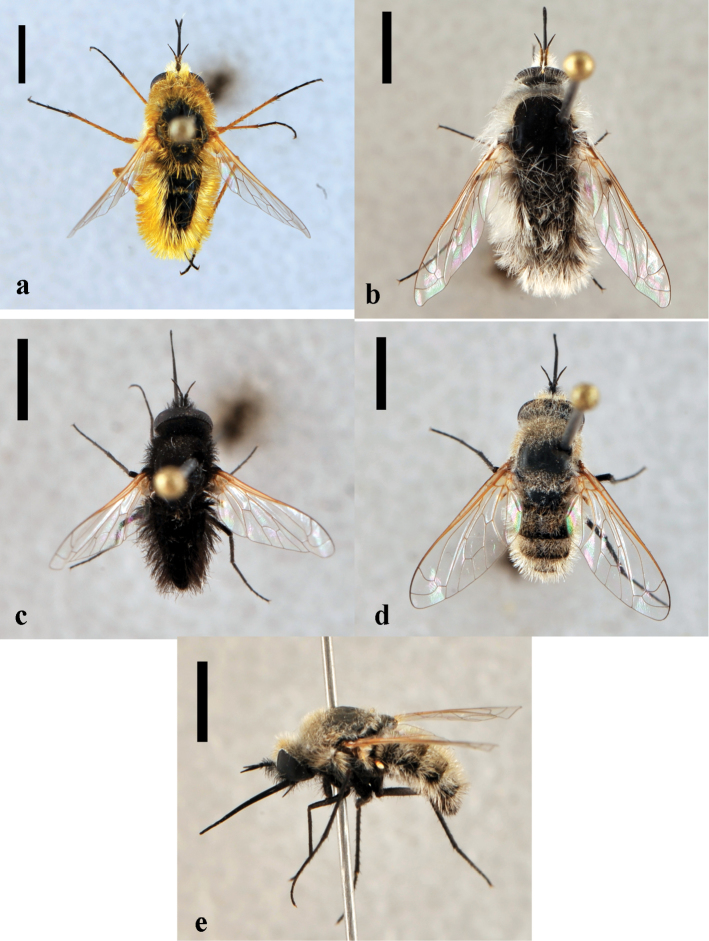
*Lordotus*. *Lordotus diplasus*, **a** female (CNIN 774) dorsal view **b** male (CNIN 861) dorsal view **c** *Lordotus divisus*, male (CNIN 777) dorsal view; *Lordotus perplexus*, female (CNIN 801) **d** dorsal view **e** lateral view. All scale bars: 3 mm.

#### Lordotus divisus

Taxon classificationAnimaliaDipteraBombyliidae

Cresson, 1919

Figure 5c

##### Material examined.

ECA: Mar (1 M), Apr (2 M); EEO: Apr (16 M); REE: Apr (4 M); ROR: Apr (1 M).

##### Known Nearctic records.

Mexico (Coahuila, Baja California); USA (Arizona, California, Nevada, New Mexico, Texas).

#### Lordotus perplexus

Taxon classificationAnimaliaDipteraBombyliidae

Johnson & Johnson, 1959

Figure 5d, e

##### Material examined.

CHU: Apr (1 H), ECA: Apr (1 H); EEO: Apr (4 F); REE: Apr (7 F); ROR: Apr (1 F).

##### Known Nearctic records.

Mexico (Baja California, Coahuila, Sinaloa, Sonora); USA (Arizona, California, Nevada, Texas).

##### Comments.

*Lordotus perplexus* has the most southern distribution in the genus, reaching Sinaloa on the Pacific coast.

#### Genus Triploechus

Taxon classificationAnimaliaDipteraBombyliidae

Edwards

##### Remarks.

Four species of *Triploechus* are present in Nearctic region: *Triploechus luridus* Hall, 1975; *Triploechus novus* (Williston, 1893); *Triploechus sackeni* (Bigot, 1892); *Triploechus stagei* Hall, 1975. Of these *Triploechus stagei* is endemic to Mexico and *Triploechus novus* has the widest distribution of this genus, being present in the south of the USA and center of Mexico. Hall and Evenhuis (1981) present a revision and key for species for this genus.

#### Triploechus novus

Taxon classificationAnimaliaDipteraBombyliidae

(Williston, 1893)

Figure 6

##### Material examined.

CHU: Apr (7 F, 6 M); REE: Apr (1 M); RPA: Apr (1 M).

##### Known Nearctic records.

Mexico (Coahuila, Durango, San Luis Potosí, Sonora); USA (Arizona, California, Nevada, New Mexico, Texas).

##### Comments.

This is a widespread and apparently common species. All specimens were collected in April so it may have a short flight season.

**Figure 6.**
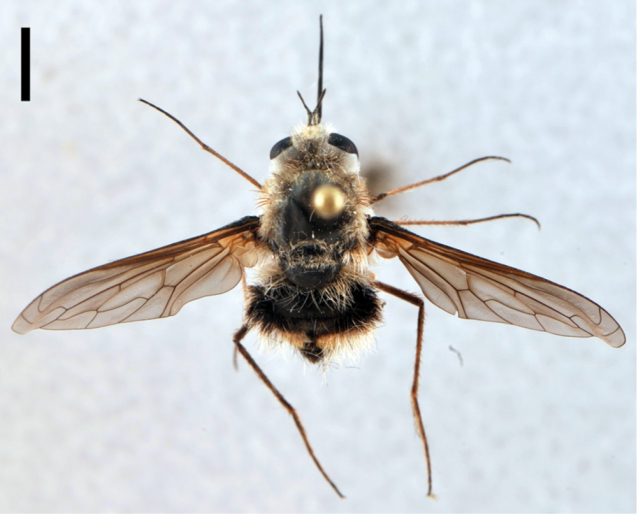
*Triploechus novus*, female (CNIN 1237) dorsal view. Scale bar: 3 mm.

### Subfamily Lomatiinae

#### Genus Ogcodocera

Taxon classificationAnimaliaDipteraBombyliidae

Macquart

##### Remarks.

The only two species in this genus have been collected from the neotropical part of Mexico to north of the USA and Canada. *Ogcodocera leucoprocta* (Wiedemann, 1828), not sampled during this study, is present in the whole Nearctic region from Canada to south of Mexico.

#### Ogcodocera analis

Taxon classificationAnimaliaDipteraBombyliidae

Williston, 1901

Figure 7

##### Material examined.

EEO: Aug (2 M), Oct (1 M).

##### Known Nearctic records.

Mexico (Coahuila, Guerrero, Morelos); USA (Arizona, Texas).

##### Comments.

This record is the first of this species in the north of Mexico, but it has been previously collected in the south of Mexico and in the south of USA, and thus is probably distributed across the whole country. Unlike *Ogcodocera leucoprocta*, *Ogcodocera analis* has its most northern distribution in Arizona and Texas.

**Figure 7.**
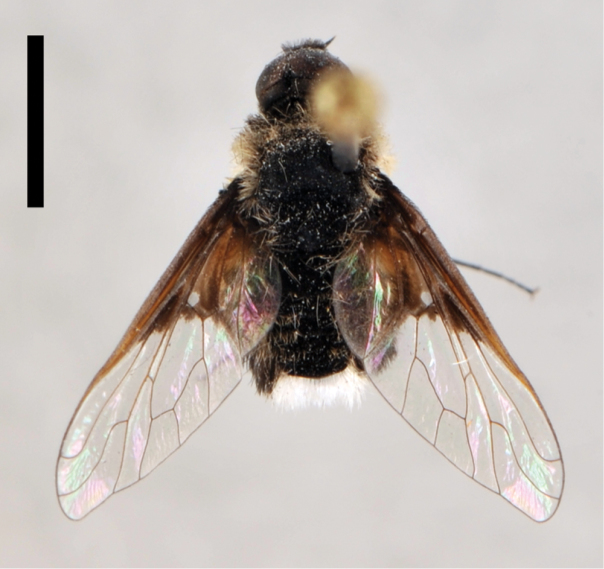
*Ogcodocera analis*, male (CNIN 146) dorsal view. Scale bar: 3 mm.

### Subfamily Tomomyzinae

#### Genus Paracosmus

Taxon classificationAnimaliaDipteraBombyliidae

Osten Sacken

##### Remarks.

All five extant species of *Paracosmus* have Nearctic distributions, and all are present in California. Two of these species have been collected in the northwest of Mexico (*Paracosmus (Actherosia) rubicundus* Melander, 1950 and *Paracosmus (Paracosmus) morrisoni* Osten Sacken, 1887).

#### Paracosmus (Paracosmus) morrisoni

Taxon classificationAnimaliaDipteraBombyliidae

Osten Sacken, 1887

Figure 8

##### Material examined.

EAM: Apr (1 F, 1 M); CHU: Apr (2 M), Jul (1 F), Aug (2 F); ECA: Apr (1 M); EEO: May (1 F); REE: Apr (1 F); ROR: Apr (2 M), May (1 F, 3 M); RPA: Apr (1 F).

##### Known Nearctic records.

Mexico (Coahuila, Sonora); USA (Arizona, California, Nevada, Texas).

##### Comments.

*Paracosmus (Paracosmus) morrisoni* has the widest distribution within this genus, but in Mexico had previously only been recorded in Sonora. This record represent the most eastern distribution for the genus in the country.

**Figure 8.**
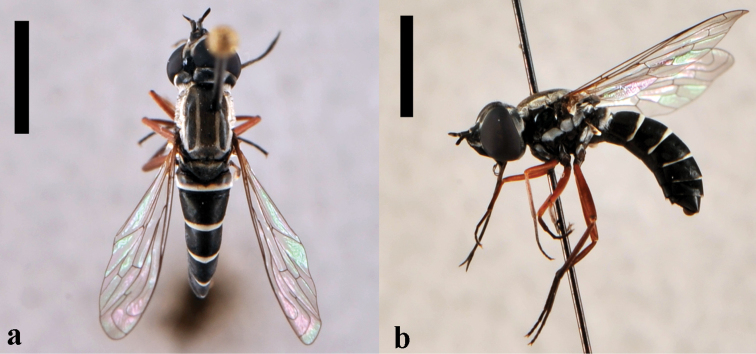
*Paracosmus (Paracosmus) morrisoni*, male (CNIN 832) **a** dorsal view **b** lateral view. All scale bars: 3 mm.

### Subfamily Anthracinae

#### Genus Anthrax

Taxon classificationAnimaliaDipteraBombyliidae

Scopoli

##### Remarks.

This is a diverse genus with 248 species worldwide. Two old but complete revisions of the genus, including distribution maps and keys, were made by Marston (1963, 1970). Thanks to these *Anthrax* species can be easily identified. Some *Anthrax* species are widely distributed occupying two biogeographic regions. From the seven *Anthrax* species collected in this study in Coahuila, just *Anthrax cybele* (Coquillett, 1894) has a restricted distribution. The other six species are widespread across the Nearctic region. Two species of *Anthrax* are reported for the first time for Mexico.

#### Anthrax atriplex

Taxon classificationAnimaliaDipteraBombyliidae

Marston, 1970

Figure 9a

##### Material examined.

EAM Apr (1 F); ROR: Oct (2 M); RPA: Aug (1 M); Sep (1 M); Oct (1 F, 2 M).

##### Known Nearctic records.

Mexico (Baja California Sur, Coahuila, Durango, Sonora, Tamaulipas); USA (Arizona, California, New Mexico, Oregon, Texas, Utah).

##### Known host.

*Megachile gentilis* Cresson (Megachilidae).

##### Comments.

This species may be present in all the north of Mexico, including Chihuahua, Nuevo León and possibly Sinaloa.

**Figure 9.**
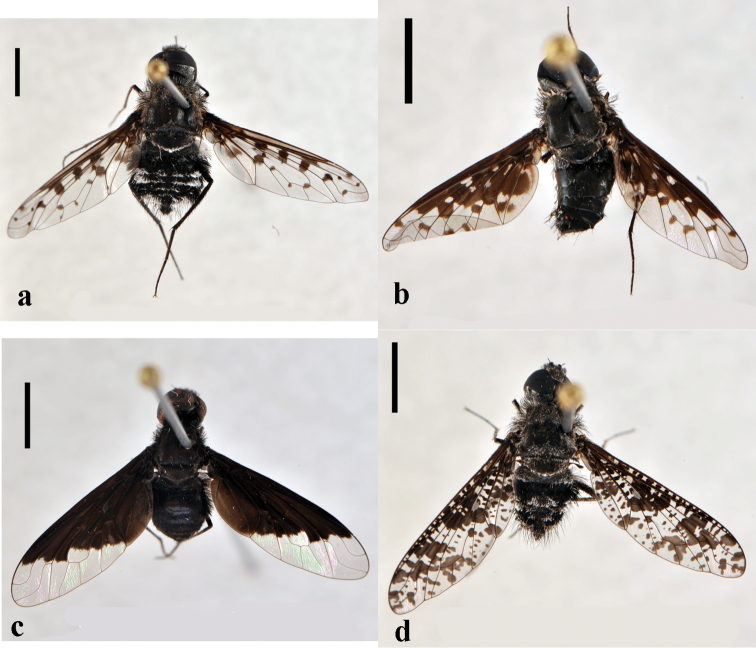
*Anthrax* part I. **a** *Anthrax atriplex*, male (CNIN 1098) dorsal view **b** *Anthrax cybele*, male (CNIN 1087) dorsal view **c** *Anthrax georgicus*, female (CNIN 1071) dorsal view **d** *Anthrax irroratus*, male (CNIN 1027) dorsal view. All scale bars: 3 mm.

#### Anthrax cybele

Taxon classificationAnimaliaDipteraBombyliidae

(Coquillett, 1894)

Figure 9b

##### Material examined.

ECA: Apr (2 F); EEO: Apr (1 M).

##### Known Nearctic records.

Mexico (Coahuila); USA (Arizona, California).

##### Comments.

This is a rare species flying in April. Its distribution is disjunct so far, present in the southwest of the USA and northeast of Mexico. It is probably also found in New Mexico and Texas in the USA and Sonora and Chihuahua in Mexico.

#### Anthrax georgicus

Taxon classificationAnimaliaDipteraBombyliidae

Macquart, 1834

Figure 9c

##### Material examined.

EAM: Mar (1 F), Apr (1 M), Jun (1 F, 2 M), Jul (2 F), Sep (2 M); ROR: Apr (1 F), Sep (1 M); RPA: Mar (1 M), Apr (1 F, 1 M), Jul (2 F, 1 M), Sep (6 F, 3 M), Oct (6 F, 3 M).

##### Known Nearctic records.

Canada (Alberta, British Columbia, Manitoba, Northwest Territory, Ontario, Quebec, Saskatchewan); Mexico (Coahuila, Guerrero, Michoacán de Ocampo, Morelos, Nuevo León, Puebla, Sonora, Veracruz); USA (Arizona, Arkansas, California, Colorado, Connecticut, Delaware, District of Columbia, Florida, Georgia, Idaho, Illinois, Iowa, Kansas, Kentucky, Maryland, Massachusetts, Michigan, Minnesota, Missouri, Montana, Nebraska, Nevada, New Hampshire, New Jersey, New Mexico, New York, North Carolina, Ohio, Oklahoma, Oregon, Pennsylvania, Tennessee, Texas, Utah, Vermont, Virginia, Washington, West Virginia, Wisconsin, Wyoming).

##### Comments.

The range of *Anthrax georgicus* includes all North America and Central America (Nicaragua, Costa Rica) covering a wide diversity of habitats and environmental conditions. Common in the rainy season and present in the dry season (March), this species is probably present in most if not all states of Mexico, but has only been collected in eight of them.

#### Anthrax irroratus

Taxon classificationAnimaliaDipteraBombyliidae

Say, 1823

Figure 9d

##### Material examined.

EAM: Apr (1 M), Aug (2 M), Oct (1 M); ECA: Apr (1 F), May (1 F, 1 M); EEO: Apr (2 F), Jul (4 F), Aug (1 M); REC: Apr (3 F, 10 M), Aug (1 M); REE: Aug (1 M); RLC: Jul (6 F, 10 M); ROR: Feb (1 M), Aug (5 M), Sep (1 M); RPA: Apr (1 M), Aug (2 M).

##### Known Nearctic records.

Canada (Alberta, British Columbia, Manitoba, Northwest Territory, Nova Scotia, Ontario, Quebec, Saskatchewan); Mexico (Baja California, Baja California Sur, Coahuila, Colima, Guerrero, Michoacán, Morelos, Nayarit, Puebla, San Luis Potosí, Sinaloa, Sonora, Veracruz, Zacatecas); USA (Alaska, Arizona, Arkansas, California, Colorado, Connecticut, Idaho, Illinois, Indiana, Kansas, Maryland, Massachusetts, Michigan, Missouri, Montana, Nebraska, Nevada, New Hampshire, New Jersey, New Mexico, New York, Oregon, Pennsylvania, Tennessee, Texas, Utah, Virginia, West Virginia, Wyoming).

##### Known hosts.

*Megachile gentilis* Cresson (Megachilidae); *Megachile mendica* Cresson (Megachilidae); *Dianthidium heterulkei fraternum* Timberlake (Megachilidae); *Aschmendiella bucconis denticulata* Cresson (Megachilidae); *Hylaeus asininus* Cockrell and Casad (Colletidae). Scott and Strickler (1992) also reared *Anthrax irroratus* from *Megachile relativa* Cresson (Megachilidae) and *Megachile inermis* Provancher (Megachilidae).

##### Comments.

*Anthrax irroratus*, like *Anthrax georgicus* (above), is present in all of North America and reaches Central America and Caribbean islands (Honduras, Puerto Rico). More abundant than its congener, this species has been collected in 15 states in Mexico (including Oaxaca of the Neotropical region not listed above) and all regions of the USA. *Anthrax irroratus* should be collected in any systematic, long term Bombyliidae sample in Mexico and the USA.

#### Anthrax oedipus

Taxon classificationAnimaliaDipteraBombyliidae

Fabricius, 1805

Figure 10a

##### Material examined.

ECA: Apr (1 F, 1 M), Jul (1 F); EEO: Apr (2 F, 1 M), May (1 F, 4 M), Jul (1 M); REC: Apr (1 F); REE: Apr (2 M); RLC: Jul (2 F, 4 M), Sep (1 F); RPA: Apr (1 M), Aug (1 M).

##### Known Nearctic records.

Mexico (Baja California, Coahuila, Nayarit, Morelos, Sinaloa, Sonora); USA (Nevada, Texas).

##### Comments.

Apparently closely related to *Anthrax irroratus*, *Anthrax oedipus* has a narrow distribution in the Nearctic region but is widely distributed in all South America. In the USA it has been collected only in two southern states, while it occurs in most of the northern states of Mexico and one central state (Morelos); it may be present in most areas from Texas to Argentina.

**Figure 10.**
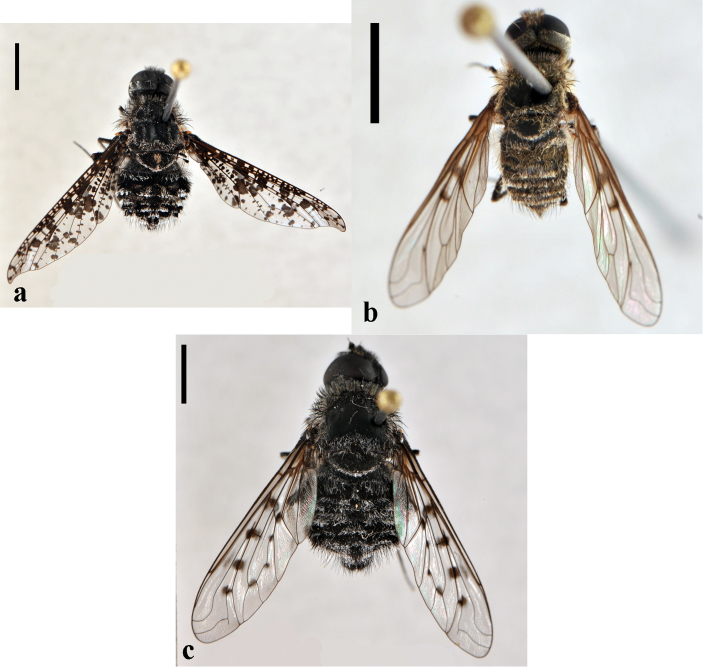
*Anthrax* part II. **a** *Anthrax oedipus*, female (CNIN 1055) dorsal view **b** *Anthrax pauper*, female (CNIN 1085) dorsal view **c** *Anthrax seriepunctatus*, female (CNIN 1089) dorsal view. All scale bars: 3 mm.

#### Anthrax pauper

Taxon classificationAnimaliaDipteraBombyliidae

(Loew, 1869)

Figure 10b

##### Material examined.

CHU: Apr (1 F, 1 M).

##### Known Nearctic records.

Canada (Ontario); Mexico (Coahuila); USA (Alabama, Colorado, Illinois, Indiana, Kansas, Maryland, Massachusetts, Michigan, Nebraska, New Jersey, New Mexico, New York, Oklahoma, Pennsylvania, Texas, Utah, Vermont, Virginia, Wisconsin).

##### Comments.

With just two specimens collected, *Anthrax pauper* appears to be a rare species in this region. This population is the most southern recorded of this species, mostly present in the center and east of the USA. Presumably adapted to colder climates, it is no coincidence that it was collected in the most elevated site sampled.

#### Anthrax seriepunctatus

Taxon classificationAnimaliaDipteraBombyliidae

(Osten Sacken, 1886b)

Figure 10c

##### Material examined.

EAM: Jun (1 M); CHU: Apr (1 F), Aug (1 F), Sep (1 F); ECA: Jun (1 M); REE: Aug (1 F); RLC: Jun (1 F), Jul (1 F, 2 M).

##### Known Nearctic records.

Mexico (Baja California Sur, Coahuila, Sonora, Puebla); USA (Arizona, Nevada, New Mexico, Texas).

##### Comments.

This species is recorded mostly from the south of the USA and north of Mexico, but its presence in Puebla in central Mexico suggests a wider distribution within the country, at least in all northern states.

#### Genus Dipalta

Taxon classificationAnimaliaDipteraBombyliidae

Osten Sacken

##### Remarks.

*Dipalta* is a small genus with just two species. *Dipalta banksi* Johnson, 1921 is only present in eastern Canada and USA, while *Dipalta serpentina* is distributed from Central America to the northern USA.

#### Dipalta serpentina

Taxon classificationAnimaliaDipteraBombyliidae

(Osten Sacken, 1877)

Figure 11

##### Material examined.

REC: Aug (1 M); RLC: Jul (1 M).

##### Known Nearctic records.

Mexico (Coahuila, Guerrero, Hidalgo, México, Morelos, Puebla, San Luis Potosí, Sinaloa); USA (Arizona, Arkansas, California, Colorado, Florida, Georgia, Idaho, Illinois, Indiana, Iowa, Kansas, Kentucky, Maine, Maryland, Massachusetts, Michigan, Minnesota, Missouri, Montana, Nebraska, Nevada, New Mexico, North Carolina, North Dakota, Ohio, Oklahoma, Oregon, South Dakota, Tennessee, Texas, Utah, Washington, Wisconsin, Wyoming).

##### Known host.

*Myrmeleon immaculatus* De Geer (Myrmeleontidae).

##### Comments.

This species is probably present in all of Mexico, but this is the only record in the north of Mexico.

**Figure 11.**
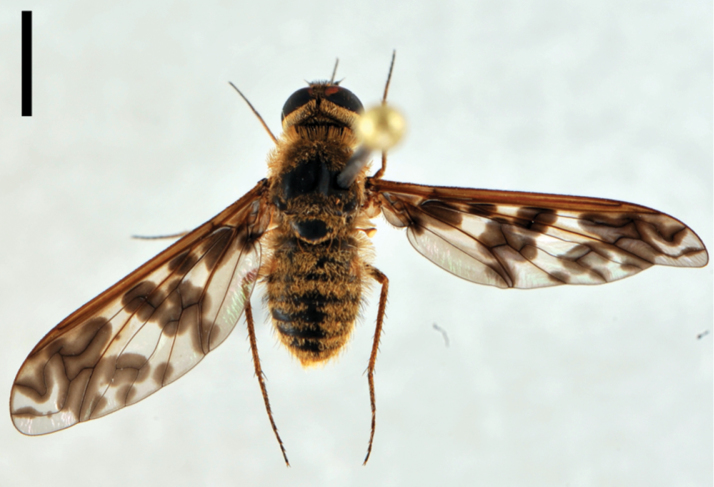
*Dipalta serpentina*, male (CNIN 215) dorsal view. Scale bar: 3 mm.

#### Genus Hemipenthes

Taxon classificationAnimaliaDipteraBombyliidae

Loew

##### Remarks.

*Hemipenthes* is equally diverse in the Nearctic (29 species), Neotropical (26 species) and Palearctic (37 species) regions, with just six species in the Oriental region and one in the Afrotropical region. Four species of this genus were collected in Coahuila. All of these have broad distributions but apparently from poor sampling because records are not continuous, especially in Mexico. Ávalos-Hernández (2009) recently published a revision of *Hemipenthes*, with a key for Nearctic species.

#### Hemipenthes jaennickeana

Taxon classificationAnimaliaDipteraBombyliidae

(Osten Sacken, 1886a)

Figure 12a

##### Material examined.

REC: Apr (18 F), Aug (4 F); REE: Feb (3 F); RLC: Mar (7 F), Jul (23 F, 3 M), Sep (3 F).

##### Known Nearctic records.

Mexico (Coahuila, Morelos, Sonora); USA (Arizona, California, Colorado, Idaho, Montana, Nevada, New Mexico, Oregon, Texas, Utah).

##### Comments.

Present mainly in the Pacific coast states of the USA and Mexico, from Oregon as far as Morelos in the center of Mexico. This record is the most eastern record in Mexico.

**Figure 12.**
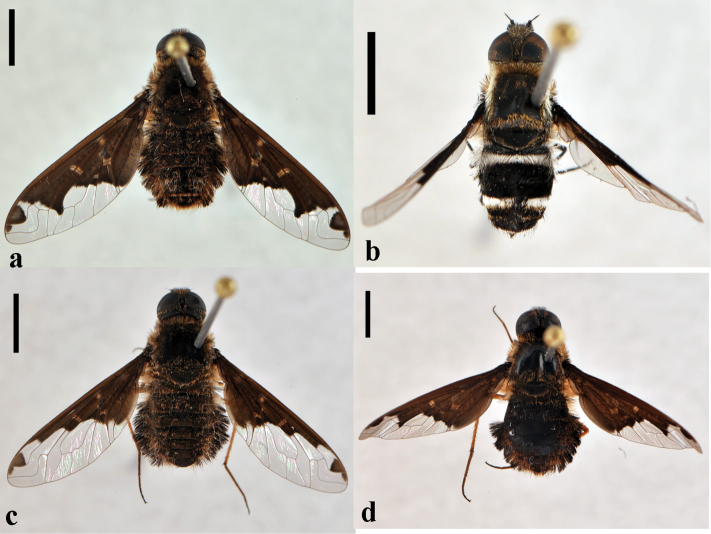
*Hemipenthes*. **a** *Hemipenthes jaennickeana*, female (CNIN 1137) dorsal view **b** *Hemipenthes lepidota*, female (CNIN 200) dorsal view **c** *Hemipenthes scylla*, male (CNIN 725) dorsal view **d** *Hemipenthes sinuosa*, female (CNIN 1134) dorsal view. All scale bars: 3 mm.

#### Hemipenthes lepidota

Taxon classificationAnimaliaDipteraBombyliidae

(Osten Sacken, 1886b)

Figure 12b

##### Material examined.

EAM: Apr (1 M), Aug (1 F); CHU: Apr (1 F, 3 M), Aug (1 M); EEO: Jul (1 F, 4 M), Aug (1 F); REC: (1 M); REE: Apr (11 F, 2 M), Aug (1 F); RLC: Jun (1 F), Jul (3 F); RPA: Apr (1 F), Sep (4 F), Oct (2 F).

##### Known Nearctic records.

Canada (Alberta); Mexico (Baja California, Baja California Sur, Coahuila, Chihuahua, Guerrero, Morelos, Puebla, San Luis Potosí, Sonora, Tamaulipas); USA (Arizona, California, Colorado, Idaho, Louisiana, Nevada).

##### Comments.

This species is abundant in the rainy season in most of the Nearctic region but has not been collected in many states of Mexico or the USA where it probably is present.

#### Hemipenthes scylla

Taxon classificationAnimaliaDipteraBombyliidae

(Osten Sacken, 1887)

Figure 12c

##### Material examined.

REC: Apr (23 M), Aug (7 M); REE: Feb (5 M), Apr (2 M); RLC: Mar (8 M), Jul (8 M), Sep (9 M).

##### Known Nearctic records.

Mexico (Coahuila, Morelos, Guanajuato, Sonora); USA (Arizona, Texas).

##### Comments.

Males of this species are abundant all year long but females are unknown. There is no explanation for this lack of females in the collections. Extreme sexual dimorphism and misidentification of females can be dismiss, since there is no *Hemipenthes* species from which only females are known. One possible explanation is that females life span is too short and therefor encounter probabilities are low. Distribution is discontinuous with populations present in central and northern Mexico and the southern USA; it is unknown whether this species is present in between these areas.

#### Hemipenthes sinuosa

Taxon classificationAnimaliaDipteraBombyliidae

(Wiedemann, 1821)

Figure 12d

##### Material examined.

REC: Apr (3 F); REE: (Feb (1 F), Apr (2 F); RLC: Jul (1 F, 1 M), Sep (1 M); RPA: Sep (2 F).

##### Known Nearctic records.

Mexico (Coahuila, Morelos); USA (Alabama, Arizona, Arkansas, Connecticut, Delaware, Georgia, Illinois, Indiana, Iowa, Kansas, Kentucky, Louisiana, Maryland, Massachusetts, Minnesota, Mississippi, Missouri, Nebraska, New Jersey, New York, North Carolina, Ohio, Oklahoma, Pennsylvania, Rhode Island, South Carolina, South Dakota, Tennessee, Texas, Vermont, Virginia, West Virginia, Wisconsin).

##### Known host.

*Neodiprion sertifer* Geoff. (Diprionidae).

##### Comments.

*Hemipenthes sinuosa* is only known from Morelos in the center of Mexico and Coahuila in the northeast, but can be found almost in all of the USA. It is clearly undersampled in Mexico.

#### Genus Lepidanthrax

Taxon classificationAnimaliaDipteraBombyliidae

Osten Sacken

##### Remarks.

Forty seven of the 52 species of *Lepidanthrax* are from the Nearctic region. Hall (1976) published a revision of this genus including keys for species.

#### Lepidanthrax arizonensis

Taxon classificationAnimaliaDipteraBombyliidae

Hall, 1976

Figure 13a

##### Material examined.

EEO: Mar (1 F); Oct (2 M).

##### Known Nearctic records.

Mexico (Coahuila); USA (Arizona).

##### Comments.

*Lepidanthrax arizonensis* has a restricted distribution, being present only in Arizona and Coahuila, but probably is also present in Chihuahua, Texas and New Mexico.

**Figure 13.**
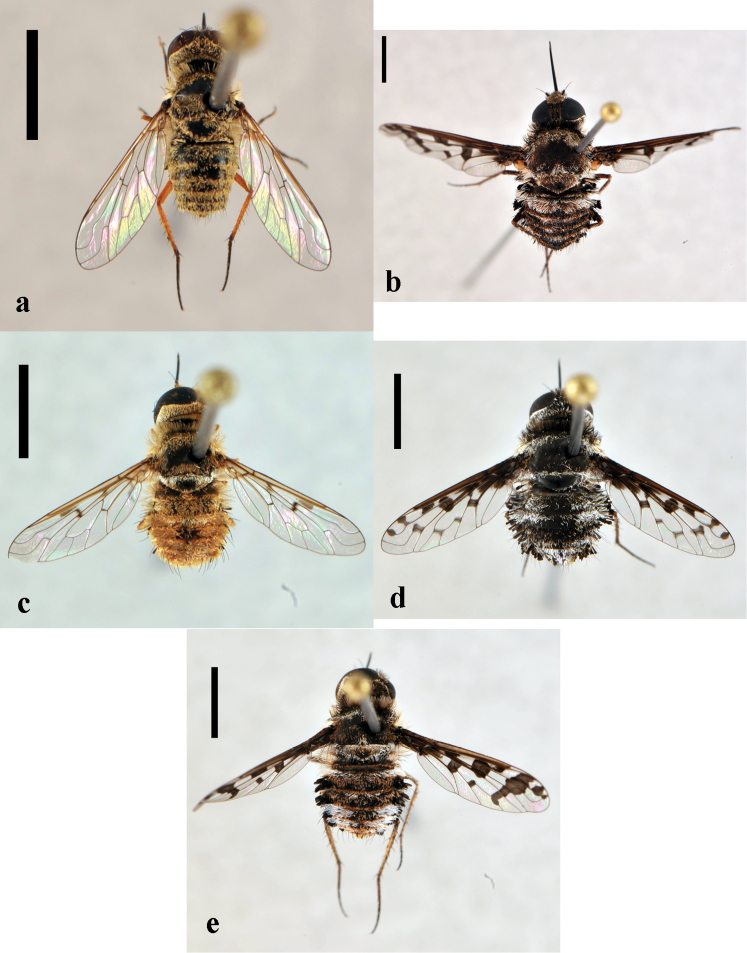
*Lepidanthrax*. **a** *Lepidanthrax arizonensis*, female (CNIN 1352) dorsal view **b** *Lepidanthrax disiunctus* female (CNIN 334) dorsal view **c** *Lepidanthrax hesperus*, male (CNIN 1339) dorsal view **d** *Lepidanthrax hyposcelus*, male (CNIN 369) dorsal view **e** *Lepidanthrax proboscideus*, male (CNIN 357) dorsal view. All scale bars: 3 mm.

#### Lepidanthrax disiunctus

Taxon classificationAnimaliaDipteraBombyliidae

(Wiedemann, 1830)

Figure 13b

##### Material examined.

REC: Aug (2 F, 1 M).

##### Known Nearctic records.

Mexico (Coahuila, Distrito Federal, Guerrero, Veracruz); USA (Arizona).

##### Comments.

The distribution of *Lepidanthrax disiunctus* has its northern extreme in Arizona and its southern extreme in Oaxaca, in the southeast of Mexico. It seems this species is rarely collected, but widely distributed.

#### Lepidanthrax hesperus

Taxon classificationAnimaliaDipteraBombyliidae

Hall, 1976

Figure 13c

##### Material examined.

EAM: Apr (2 M); CHU: Apr (2 F, 5 M); ROR: Apr (1 F, 3 M), May (1 F, 1 M); RPA: Apr (1 F, 14 M).

##### Known Nearctic records.

Mexico (Baja California, Coahuila, Sinaloa, Sonora); USA (Arizona, California, New Mexico, Texas).

##### Comments.

This record is the first in northeastern Mexico.

#### Lepidanthrax hyposcelus

Taxon classificationAnimaliaDipteraBombyliidae

Hall, 1976

Figure 13d

##### Material examined.

RLC: Sep (4 F, 15 M).

##### Known Nearctic records.

Mexico (Coahuila, Guerrero, Morelos, Puebla).

##### Comments.

*Lepidanthrax hyposcelus* is endemic to Mexico, previously only known from the southwest of the country; this record extends its distribution to the northeast of the country.

#### Lepidanthrax proboscideus

Taxon classificationAnimaliaDipteraBombyliidae

(Loew, 1869)

Figure 13e

##### Material examined.

ECA: Sep (1 F, 2 M); EEO: Apr (1 F), Aug (1 F, 1 M), Oct (4 F, 15 M); ROR: Sep (2 M); RPA: Sep (2 M), Oct (1 M).

##### Known Nearctic records.

Mexico (Baja California, Baja California Sur, Coahuila, Durango, Guerrero, Morelos, Sonora); USA (Arizona, California, Nevada, New Mexico, Utah).

##### Comments.

*Lepidanthrax proboscideus*, *Lepidanthrax fuscipennis* Hall, 1976 and *Lepidanthrax disiunctus* are the only species of this genus distributed in both the Nearctic and Neotropical regions. Of these *Lepidanthrax proboscideus* extends as far as El Salvador, the most southern distribution for a Nearctic species of this genus. This is the first record of this species in the northeast of Mexico.

#### Genus Neodiplocampta

Taxon classificationAnimaliaDipteraBombyliidae

Curran

##### Remarks.

*Neodiplocampta* is a small American genus, more diverse in the Neotropical than the Nearctic region. Hull and Martin (1974) described seven of the 16 species and published a key for all species of the genus.

#### Neodiplocampta (Neodiplocampta) miranda

Taxon classificationAnimaliaDipteraBombyliidae

Hull & Martin, 1974

Figure 14

##### Material examined.

CHU: Aug (1 F); EEO: Aug (1 F, 1 M); ROR: Jul (1 F); Oct (1 M); RPA: Aug (1 F, 2 M).

##### Known Nearctic records.

Mexico (Coahuila, Guerrero, San Luis Potosi, Sinaloa, Sonora); USA (Arizona, California, Florida, Texas).

##### Comments.

*Neodiplocampta (Neodiplocampta) miranda* and *Neodiplocampta (Agitonia) sepia* Hull, 1966 are the only two species distributed in both biogeographic regions (Nearctic and Neotropical). This species is distributed from the south of the USA to Nicaragua, but has not been collected in most Mexican states. This lack of records is possibly due its low abundance.

**Figure 14.**
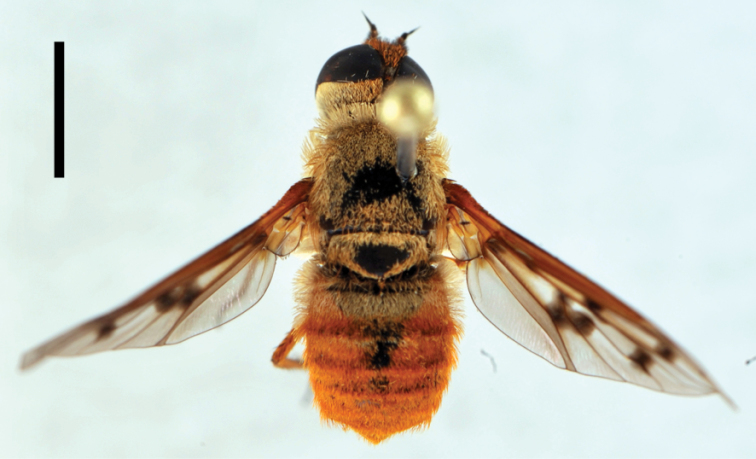
*Neodiplocampta (Neodiplocampta) miranda*, female (CNIN 225) dorsal view. Scale bar: 3 mm.

#### Genus Paravilla

Taxon classificationAnimaliaDipteraBombyliidae

Painter

##### Remarks.

Fifty five of the 58 species of the genus are Nearctic. All species of *Paravilla* collected in Coahuila were exclusively collected in the summer months from April to July. Hall (1981a) reviewed this genus and presented a key for species and description of new species.

#### Paravilla edititoides

Taxon classificationAnimaliaDipteraBombyliidae

(Painter, 1933)

Figure 15a

##### Material examined.

EAM: Jun (1 M); CHU: Apr (1 F), Jul (2 F, 1 M); ECA: Apr (1 F, 1 M), Jul (1 M); EEO: Apr (1 F, 10 M), May (1 F, 2 M), Jul (9 M); REE: Apr (1 M); RLC: Jun (2 F, 5 M), Jul (1 F); ROR: Jul (1 F, 3 M); RPA: Oct (1 M).

##### Known Nearctic records.

Canada (Saskatchewan); Mexico (Chihuahua, Coahuila, Durango, Jalisco, México, Zacatecas); USA (Arizona, Colorado, Idaho, Kansas, Montana, Nebraska, New Mexico, Oklahoma, Utah, Texas, Wyoming).

##### Comments.

This species is very abundant and present in most of North America, from Canada as far as Jalisco in central Mexico.

**Figure 15.**
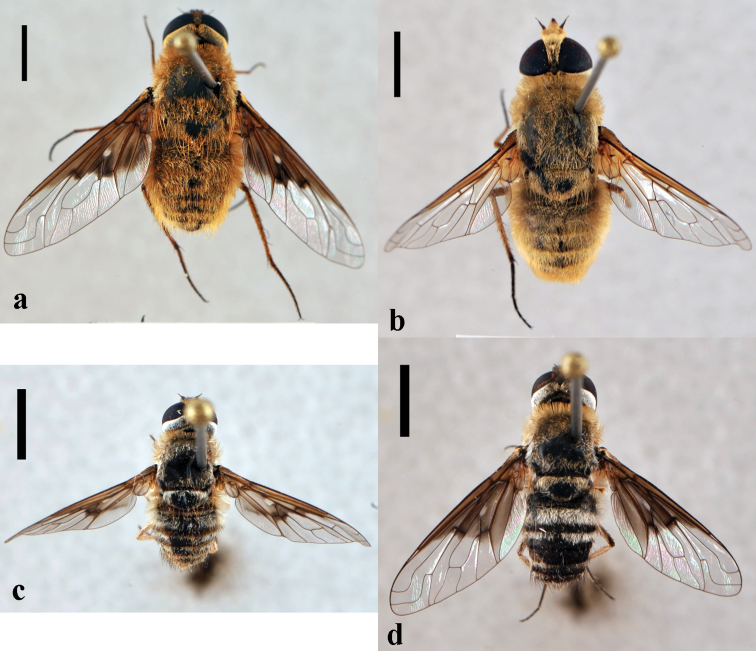
*Paravilla*. **a** *Paravilla edititoides*, male (CNIN 1272) dorsal view **b** *Paravilla flavipilosa*, male (CNIN 1125) dorsal view **c** *Paravilla parvula*, female (CNIN 884) dorsal view **d** *Paravilla separata*, female (CNIN 898) dorsal view. All scale bars: 3 mm.

#### Paravilla flavipilosa

Taxon classificationAnimaliaDipteraBombyliidae

(Cole, 1923)

Figure 15b

##### Material examined.

CHU: Apr (1 M); Jul (1 M); ECA: Apr (1 M); EEO: Apr (7 M), May (11 M); ROR: Apr (2 M); RPA: Apr (1 M).

##### Known Nearctic records.

Mexico (Baja California Sur, Coahuila, Nuevo León); USA (Arizona, California, Colorado, Texas).

##### Comments.

*Paravilla flavipilosa* is abundant and restricted to the south of the USA and north of Mexico.

#### Paravilla parvula

Taxon classificationAnimaliaDipteraBombyliidae

Hall, 1981a

Figure 15c

##### Material examined.

EAM: Apr (1 F); CHU: Apr (1 M); RPA: Apr (7 F, 13 M).

##### Known Nearctic records.

Mexico (Chihuahua, Coahuila, Durango, Guanajuato, Hidalgo, Jalisco, México, Michoacán, Nuevo León, San Luis Potosí, Sonora, Zacatecas), USA (Arizona, New Mexico, Texas, Utah).

##### Comments.

*Paravilla parvula* is relatively well collected in northern and central Mexico. Its distribution also includes the south of the USA but no farther north than Utah.

#### Paravilla separata

Taxon classificationAnimaliaDipteraBombyliidae

(Walker, 1852)

Figure 15d

##### Material examined.

CHU: Apr (1 F); EEO: Apr (3 F); REE: Apr (5 F, 3 M); RPA: Apr (1 M).

##### Known Nearctic records.

Canada (Ontario, Manitoba); Mexico (Coahuila); USA (Alabama, Florida, Georgia, Iowa, Kansas, Michigan, Minnesota, Mississippi, Nebraska, Ohio, South Dakota, Wisconsin).

##### Comments.

*Paravilla separata* is present mainly in the eastern half of the USA, and southeastern Canada. This record in Coahuila represents the southern extreme of the distribution of this species, and is the first in Mexico. It may also be present in Tamaulipas and Nuevo León but doubtfully in the northwest of Mexico.

#### Genus Poecilanthrax

Taxon classificationAnimaliaDipteraBombyliidae

Osten Sacken

##### Remarks.

Four species from this mainly Nearctic genus are recorded in Coahuila for the first time. Painter and Hall (1960) published a review of *Poecilanthrax* with a key and images of the species.

#### Poecilanthrax effrenus

Taxon classificationAnimaliaDipteraBombyliidae

(Coquillett, 1887)

Figure 16a

##### Material examined.

EAM: Apr (1 F, 1 M), Jun (1 F, 1 M), Sep (1 F); CHU: Jun (1 F); ROR: May (10 F, 5 M), Jul (2 F, 3 M), Aug (1 M); RPA: Jun (4 F, 1 M), Jul (6 F, 6 M), Aug (1 F, 1 M), Sep (4 F), Oct (2 F, 1 M).

##### Known Nearctic records.

Mexico (Baja California Sur, Chihuahua, Coahuila, Sonora, Tamaulipas); USA (Arizona, California, New Mexico, Oklahoma, Texas).

##### Comments.

This record fills a gap in *Poecilanthrax effrenus* distribution between northwest and northeast populations of Mexico. This species is probably present in Baja California and Nuevo León, but has not yet been recorded.

**Figure 16.**
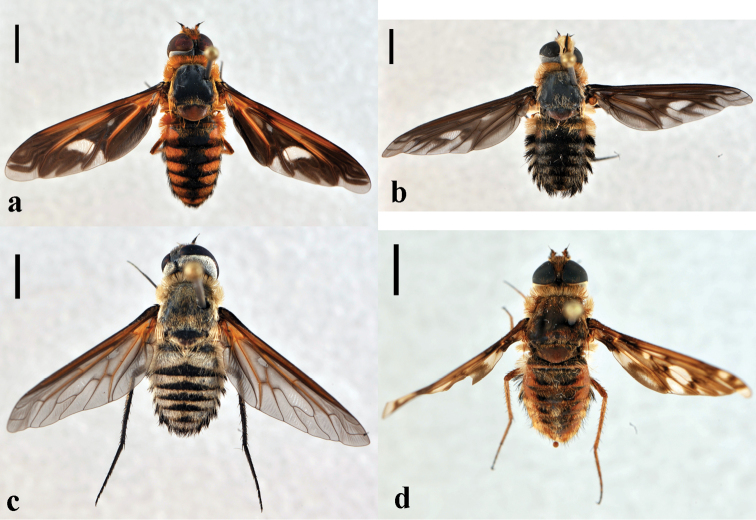
*Poecilanthrax*. **a** *Poecilanthrax effrenus*, female (CNIN 1380) dorsal view **b** *Poecilanthrax fasciatus*, male (CNIN 218) dorsal view **c** *Poecilanthrax hyalinipennis*, female (CNIN 1365) dorsal view **d** *Poecilanthrax poecilogaster*, male (CNIN 1356) dorsal view. All scale bars: 3 mm.

#### Poecilanthrax fasciatus

Taxon classificationAnimaliaDipteraBombyliidae

Johnson & Johnson, 1957

Figure 16b

##### Material examined.

EAM: Sep (1 M); CHU: Oct (1 M); ROR: Oct (1 M); RPA: Oct (1 M).

##### Known Nearctic records.

Mexico (Coahuila); USA (Colorado, Kansas, Texas).

##### Known host.

*Chorizagrotis auxiliaris* Grote (Noctuidae).

##### Comments.

*Poecilanthrax fasciatus* is collected in Mexico for the first time, and this extends the southern limit of this species distribution.

#### Poecilanthrax hyalinipennis

Taxon classificationAnimaliaDipteraBombyliidae

Painter & Hall, 1960

Figure 16c

##### Material examined.

EAM: Mar (3 M), Oct (1 F); CHU: Oct (1 M); ROR: Oct (4 M); RPA: Sep (1 F), Oct (2 F, 6 M).

##### Known Nearctic records.

Mexico (Coahuila); USA (Arizona, California, Nevada, Utah).

##### Comments.

This record extends the distribution of *Poecilanthrax hyalinipennis* into the northwest of Mexico. Considering its distribution in the USA, this species may also be present in the northeast of Mexico.

#### Poecilanthrax poecilogaster

Taxon classificationAnimaliaDipteraBombyliidae

(Osten Sacken, 1886b)

Figure 16d

##### Material examined.

REE: Apr (2 M).

##### Known Nearctic records.

Canada (Alberta, Manitoba, Ontario, Saskatchewan); Mexico (Coahuila, Morelos, Nuevo León, Sonora); USA (Arizona, California, Colorado, Idaho, Nevada, New Mexico, Oregon, Utah).

##### Comments.

Most of the records in the USA and Mexico of this rarely collected but widespread species are from Pacific Coast states, although, there are records from Nuevo Leon and Coahuila in northeast Mexico.

#### Genus Rhynchanthrax

Taxon classificationAnimaliaDipteraBombyliidae

Painter

##### Remarks.

Of the seven species of this exclusively Nearctic genus, six are present in Mexico, with *Rhynchanthrax maria* (Williston, 1901) and *Rhynchanthrax nigrofimbriatus* (Williston, 1901) being endemic to this country. Only *Rhynchanthrax parvicornis* (Loew, 1869) has not been collected in Mexico, but it is distributed across the southern USA and may also occur in the north of Mexico.

#### Rhynchanthrax capreus

Taxon classificationAnimaliaDipteraBombyliidae

(Coquillett, 1887)

Figure 17a

##### Material examined.

CHU: Apr (1 M), Aug (13 F, 6 M), Jul (12 F, 6 M), Sep (1 F, 3 M).

##### Known Nearctic records.

Mexico (Coahuila); USA (Arizona, California, Colorado, Nebraska, Nevada, New Mexico, Utah).

##### Comments.

This is the first record of this species in Mexico. *Rhynchanthrax capreus* is the only species occurring in the northwest of the USA, while the other species in the genus are present mainly in the south and east of the country. This species may also be present in the northwest of Mexico (Baja California, Sonora, Chihuahua).

**Figure 17.**
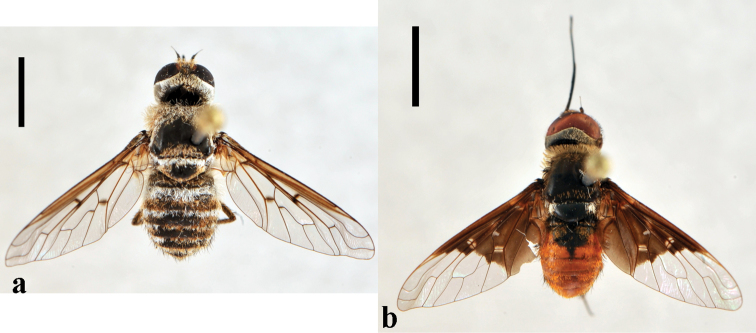
*Rhynchanthrax*. **a** *Rhynchanthrax capreus*, female (CNIN 940) dorsal view **b** *Rhynachantrax texanus*, male (CNIN 263) dorsal view. All scale bars: 3 mm.

#### Rhynachantrax texanus

Taxon classificationAnimaliaDipteraBombyliidae

(Painter, 1933)

Figure 17b

##### Material examined.

EEO: May (1 M); RLC: Jun (1 F, 11 M), Jul (3 M).

##### Known Nearctic records.

Mexico (Coahuila, Sonora); USA (Kansas, New Mexico, Texas).

##### Comments.

This is the most eastern record in Mexico for this species. In the USA it is distributed in the southern-center of the country, but in Mexico it has been collected in Sonora so it probably also occurs in Arizona.

#### Genus Thyridanthrax

Taxon classificationAnimaliaDipteraBombyliidae

Osten Sacken

##### Remarks.

*Thyridanthrax* has twice as many species in the Palearctic region as in the Nearctic and Neotropical regions combined. All 12 species in North America are present in the USA with five also in Mexico. These are the first records of this genus in Coahuila. The distribution of *Thyridanthrax selene* (Osten Sacken, 1886b) and *Thyridanthrax pallidus* (Coquillett, 1887) are very similar, being present in all of the southern USA and probably also in all of northern Mexico, although they have been only collected in Sonora and Coahuila to date. Both species are rare and were collected only in April.

#### Thyridanthrax pallidus

Taxon classificationAnimaliaDipteraBombyliidae

(Coquillett, 1887)

Figure 18b

##### Material examined.

REE: Apr (4 F, 1 M).

##### Known Nearctic records.

Mexico (Coahuila); USA (Arizona, California, Nevada, Texas, Utah).

##### Comments.

This represents the first record of this species in Mexico.

**Figure 18.**
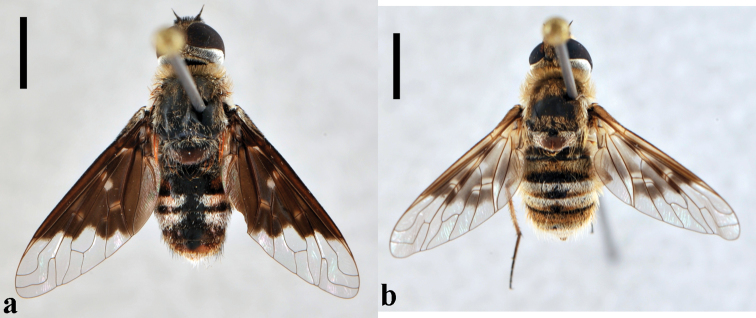
*Thyridanthrax*. **a** *Thyridanthrax selene*, male (CNIN 182) dorsal view **b** *Thyridanthrax pallidus*, female (CNIN 1162) dorsal view. All scale bars: 3 mm.

#### Thyridanthrax selene

Taxon classificationAnimaliaDipteraBombyliidae

(Osten Sacken, 1886b)

Figure 18a

##### Material examined.

EAM: Apr (1 M); REE: Apr (2 F, 2 M).

##### Known Nearctic records.

Mexico (Coahuila, Sonora); USA (Arizona, California, Texas).

##### Comments.

This is the most eastern record in Mexico.

#### Genus Xenox

Taxon classificationAnimaliaDipteraBombyliidae

Evenhuis

##### Remarks.

Of the five species that constitute this genus, four are present in Mexico.

#### Xenox xylocopae

Taxon classificationAnimaliaDipteraBombyliidae

(Marston, 1970)

Figure 19

##### Material examined.

ECA: Sep (1 M).

##### Known Nearctic records.

Mexico (Chihuahua, Coahuila, Sonora), USA (Arizona, New Mexico, Texas).

##### Known host.

*Xylocopa micheneri micheneri* (Hurd) (Apidae) as reported by Minckley (1989).

##### Comments.

*Xenox xylocopae* appears to be restricted to the northeast of Mexico and south of the USA. Three of the other species also have restricted and separate distributions: *Xenox delila* Loew, 1869 is present in the northwest of Mexico and California; *Xenox nigritus* (Schaeffer, 1768) occurs from the northeast of Mexico (Veracruz and Tamaulipas without overlap with *Xenox xylocopae*) to South America; and *Xenox tigrinus* (De Geer, 1776) is present in the eastern USA and southern Ontario. Only *Xenox habrosus* (Marston, 1970) has a distribution overlapping with the other four species, being present in all of Mexico and the southwest of the USA.

**Figure 19.**
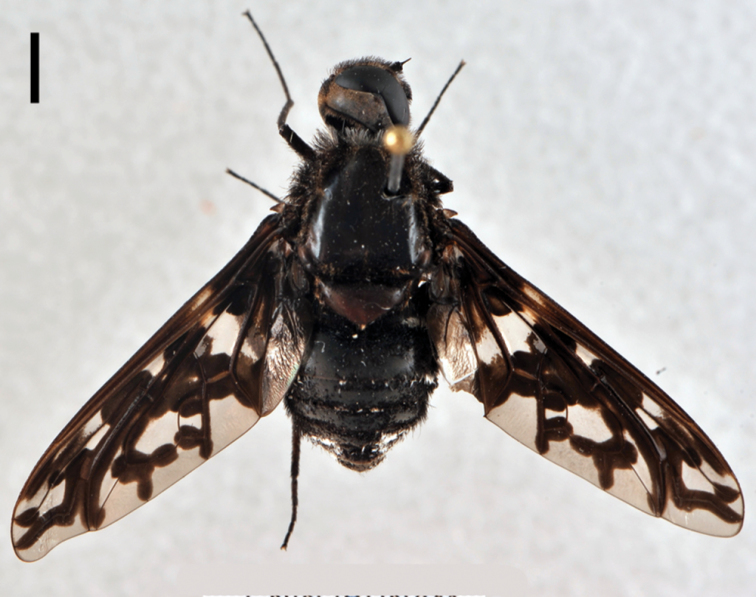
*Xenox xylocopae*, male (CNIN 1165) dorsal view. Scale bar: 3 mm.

## Discussion

The data presented here increase the knowledge of Bombyliidae in Mexico but also reveals the deficiencies in sampling of the family in the country. The species list for the state increased three-fold, which demonstrates the lack of knowledge of the Bombyliidae fauna in this region. Almost all states of Mexico are in a similar situation but northern states appear to have higher diversity and should be priorities for sampling. Hull (1973) identified the northwest of Mexico as a species concentration area of Bombyliidae, but the northeast portion of the country may have the same species richness. Diversity of this family in the north of Mexico is probably much higher than recorded, as indicated by the richness in the south of USA which has similar environmental characteristics but much better sampling. Therefore northeast Mexico is possibly one of the most under sampled areas in the Nearctic region for Bombyliidae, given the great diversity of this family in the area, combined with the size of this part of the country. The study of Bombyliidae in the northern states of Mexico should be more of a priority than field work in the center or the southern states.

Most of the species collected in this study have a broad distribution in the USA but Mexican records are isolated. There are probably more species yet to be recorded from Coahuila and other Mexican states, especially species present in southern border states of the USA. Some species are recorded only from Coahuila in the northeast of Mexico but are also present in the northwest of the country. More studies are required to determine if these species have a disjunct distribution or if any are represented by distinct, cryptic eastern and western species.

Cuatro Ciénegas' biological and conservational importance has long been recognized for reptiles (McCoy 1984), birds (Contreras-Balderas 1984), plants (Pinkava 1984, Villarreal and Encina 2005), snails (Hershler 1984), Crustacea (Cole 1984) and particularly fishes (Minckley 1984), but little is known of other groups like insects. The insects contain 53% of the described species in the planet (Chapman 2009), so their distribution and diversity should be considered for conservation and natural reserve design. The diversity of insects, especially of Bombyliidae and similar arid-regions-diverse groups, increases the conservational value of Cuatro Ciénegas.

## Conclusions

The data presented here indicates the significance of Cuatro Ciénegas for Bombyliidae diversity. Comparison with other nearby areas should be undertaken to confirm whether this area really is richer for this family. Data also reveal that true species richness of Bombyliidae is much higher than previously recorded. This could also be true for other insect groups. More funding should be destined for faunistic studies of megadiverse groups with ecological importance such as Diptera, Coleoptera, Hymenoptera and Lepidoptera. The information obtained from these studies might be used first to quantify the species richness and species exchange between areas (beta diversity) (Whittaker 1972) and later to propose conservation management schemes.

## Supplementary Material

XML Treatment for Toxophora

XML Treatment for Toxophora maxima

XML Treatment for Toxophora virgata

XML Treatment for Bombylius

XML Treatment for Bombylius (Bombylius) frommerorum

XML Treatment for Heterostylum

XML Treatment for Heterostylum croceum

XML Treatment for Lordotus

XML Treatment for Lordotus diplasus

XML Treatment for Lordotus divisus

XML Treatment for Lordotus perplexus

XML Treatment for Triploechus

XML Treatment for Triploechus novus

XML Treatment for Ogcodocera

XML Treatment for Ogcodocera analis

XML Treatment for Paracosmus

XML Treatment for Paracosmus (Paracosmus) morrisoni

XML Treatment for Anthrax

XML Treatment for Anthrax atriplex

XML Treatment for Anthrax cybele

XML Treatment for Anthrax georgicus

XML Treatment for Anthrax irroratus

XML Treatment for Anthrax oedipus

XML Treatment for Anthrax pauper

XML Treatment for Anthrax seriepunctatus

XML Treatment for Dipalta

XML Treatment for Dipalta serpentina

XML Treatment for Hemipenthes

XML Treatment for Hemipenthes jaennickeana

XML Treatment for Hemipenthes lepidota

XML Treatment for Hemipenthes scylla

XML Treatment for Hemipenthes sinuosa

XML Treatment for Lepidanthrax

XML Treatment for Lepidanthrax arizonensis

XML Treatment for Lepidanthrax disiunctus

XML Treatment for Lepidanthrax hesperus

XML Treatment for Lepidanthrax hyposcelus

XML Treatment for Lepidanthrax proboscideus

XML Treatment for Neodiplocampta

XML Treatment for Neodiplocampta (Neodiplocampta) miranda

XML Treatment for Paravilla

XML Treatment for Paravilla edititoides

XML Treatment for Paravilla flavipilosa

XML Treatment for Paravilla parvula

XML Treatment for Paravilla separata

XML Treatment for Poecilanthrax

XML Treatment for Poecilanthrax effrenus

XML Treatment for Poecilanthrax fasciatus

XML Treatment for Poecilanthrax hyalinipennis

XML Treatment for Poecilanthrax poecilogaster

XML Treatment for Rhynchanthrax

XML Treatment for Rhynchanthrax capreus

XML Treatment for Rhynachantrax texanus

XML Treatment for Thyridanthrax

XML Treatment for Thyridanthrax pallidus

XML Treatment for Thyridanthrax selene

XML Treatment for Xenox

XML Treatment for Xenox xylocopae
